# A new method for quantifying mitochondrial axonal transport

**DOI:** 10.1007/s13238-016-0268-3

**Published:** 2016-05-25

**Authors:** Mengmeng Chen, Yang Li, Mengxue Yang, Xiaoping Chen, Yemeng Chen, Fan Yang, Sheng Lu, Shengyu Yao, Timothy Zhou, Jianghong Liu, Li Zhu, Sidan Du, Jane Y. Wu

**Affiliations:** 1 University of Chinese Academy of Sciences, Beijing, 100049 China; 2 School of Electronic Science & Engineering, Nanjing University, Nanjing, 210093 China; 3 State Key Laboratory for Brain & Cognitive Science, Institute of Biophysics, Chinese Academy of Sciences, Beijing, 100101 China; 4 Department of Neurology, Center for Genetic Medicine, Lurie Cancer Center, Northwestern University Feinberg School of Medicine, Chicago, IL 60611 USA

**Keywords:** mitochondrial transport, image processing and analysis, FUS proteinopathy and mitochondrial transport defect

## Abstract

Axonal transport of mitochondria is critical for neuronal survival and function. Automatically quantifying and analyzing mitochondrial movement in a large quantity remain challenging. Here, we report an efficient method for imaging and quantifying axonal mitochondrial transport using microfluidic-chamber-cultured neurons together with a newly developed analysis package named “MitoQuant”. This tool-kit consists of an automated program for tracking mitochondrial movement inside live neuronal axons and a transient-velocity analysis program for analyzing dynamic movement patterns of mitochondria. Using this method, we examined axonal mitochondrial movement both in cultured mammalian neurons and in motor neuron axons of Drosophila *in vivo*. In 3 different paradigms (temperature changes, drug treatment and genetic manipulation) that affect mitochondria, we have shown that this new method is highly efficient and sensitive for detecting changes in mitochondrial movement. The method significantly enhanced our ability to quantitatively analyze axonal mitochondrial movement and allowed us to detect dynamic changes in axonal mitochondrial transport that were not detected by traditional kymographic analyses.

**Electronic supplementary material:**

The online version of this article (doi:10.1007/s13238-016-0268-3) contains supplementary material, which is available to authorized users.

## INTRODUCTION

Mitochondria act as both critical powerhouse and important signaling station(s) in eukaryotic cells (for recent reviews see Wang and Schwarz, 2009a; Wallace 2013). Moving along microtubules, mitochondria are transported by molecular motors. All eukaryotic cells depend on mitochondria for local ATP supply and calcium buffering (Rizzuto et al., 1998; Wang and Schwarz, 2009b). In neurons, active cargo transport between the cell body and neuronal processes (neurites) is essential for proper distribution of critical materials and energy to their respective subcellular locations (Radad et al., 2006; Singh et al., 2014). Such cargo transport depends on mitochondria. Defective mitochondrial biogenesis and transport have been associated with a wide range of neurodegenerative diseases, including amyotrophic lateral sclerosis (ALS), Parkinson’s disease and Alzheimer’s disease (Hollenbeck and Grabensee, 1993; Liu et al., 2012; Mattson et al., 2008; Reddy, 2011; Reddy and Shirendeb, 2012; Reeve et al., 2008; Salinas et al., 2008; Sheng and Cai, 2012; Yadav et al., 2014).

Significant advance has been made in mitochondrial biology in the past few decades. Driven by molecular motors, mitochondria exhibits complex and characteristic patterns of movement, traveling back and forth with numerous starts and stops along microtubules (Chan, 2006; Hollenbeck and Saxton, 2005; Kann and Kovacs, 2007; Lin and Sheng, 2015; Morris and Hollenbeck, 1995; Overly et al., 1996). Nonetheless, our ability to image and quantify axonal mitochondrial transport remains limited, although recent studies have begun to provide insights into these important processes (Ashrafi et al., 2014; Hollenbeck and Grabensee, 1993; Overly and Hollenbeck, 1996; Zhang et al., 2011). Several factors make the task of high-throughput imaging of mitochondrial movement particularly difficult. First, variation in mitochondrial morphology makes it difficult to efficiently segment and identify mitochondria. Second, photo bleaching forces a compromise between frame rate and duration of tracking time (Yang et al., 2010). Finally, the low signal-to-noise ratio (SNR) in mitochondrial imaging has been a major roadblock for developing efficient tracking algorithm (Yang et al., 2012). Efficient methods remain to be developed for automatically tracking and quantitatively analyzing mitochondrial movement.

To study the axonal transport process, it is important to follow the trajectories of individual axonal cargos over time. Generating kymographs from time-lapse images is perhaps the most frequently used technique in studying mitochondrial axonal transport (e.g., Miller and Sheetz, 2004; Hollenbeck and Saxton, 2005; references within Wang et al., 2011). In this type of analysis, indices such as instantaneous velocity, run time in both directions (anterograde versus retrograde) have been used to characterize mitochondrial movement. These parameters can be determined by using general-purpose image processing software. In some studies, Matlab-based programs were developed to achieve automatic analyses (Wang and Schwarz., 2009b). These published methods have three major limitations. First, the lack of an efficient imaging method for mitochondrial movement resulted in the labor-intensive and time-consuming nature of the published methods. Second, insufficient sample sizes limited the reliability and sensitivity of published analysis programs. Third, mathematical models for describing and quantifying mitochondrial movement patterns were not established. These limitations made the published methods inadequate for high-throughput analyses (Bros et al., 2015). Here we report a new method for studying the dynamic progress of axonal mitochondrial transport by coupling a highly efficient imaging method with automated mitochondrial tracking and motion pattern analysis algorithms.

## RESULTS

### Imaging axonal mitochondrial movement in a high-throughput manner

**Figure 1:**
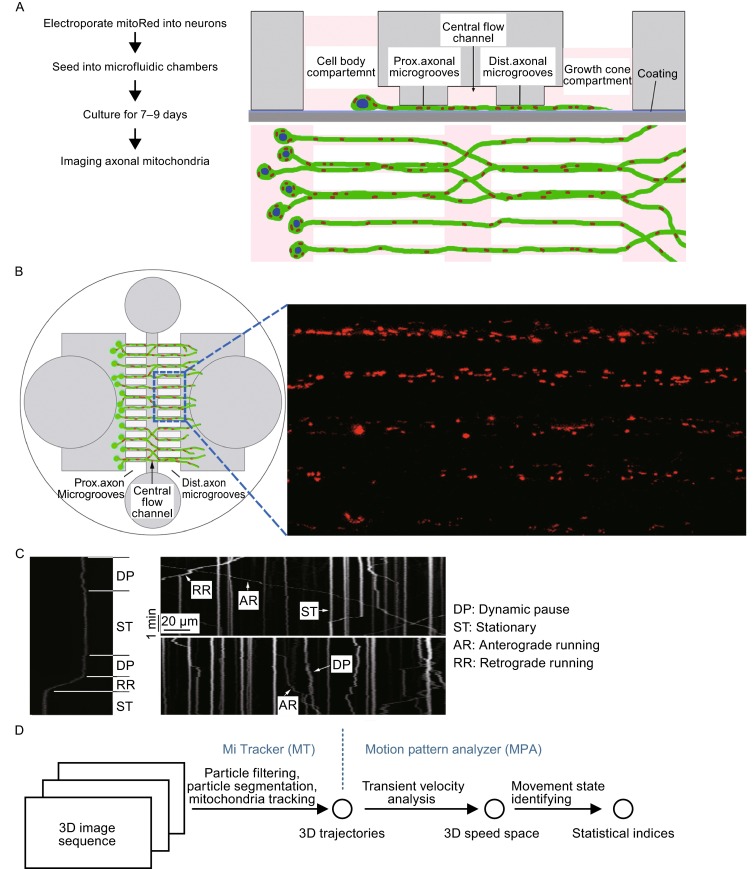
MitoQuant: a toolkit for analyzing axonal mitochondrial transport. (A and B) A microfluidic chamber was used to culture cortical neurons, making it possible to image axonal mitochondrial transport in a high throughput manner. (A) A flow chart of neuronal culture in microfluidic chambers, together with the side view (top panel) and top view (bottom panel) of chambers containing neurons expressing mitoRed to mark mitochondria for fluorescent confocal microscopic imaging. (B) A diagram to illustrate the microfluidic neuronal culture (left) and a fluorescent confocal microscopic image of axonal mitochondria (right), showing that >100 mitochondria distributed in 5 axonal bundles inside axonal microgrooves can be captured in one series of confocal imaging. An example of bright field image of axonal bundles is shown in Fig. S7. (C) 2-D Kymographs of axonal mitochondria in 5 min to demonstrate different movement states of mitochondria. Scale bars: 1 min and 20 μm. Representative illustration of different mitochondrial movement states: stationary (ST), dynamic pause (DP), anterograde running (AR) and retrograde running (RR), as marked in the kymographs. (D) The analysis toolkit consists of two image-analysis programs: MiTracker (MT) for tracking mitochondria by locating mitochondria and linking their coordinates into 3-D trajectories and motion pattern analyzer (MPA) for identifying the mitochondrial movement states in a 2-D speed space (transient and sustained speed)

To efficiently image axonal mitochondria, we developed a modified microfluidic system for neuronal culture following labeling mitochondria with mitochondrial localized Red Fluorescent protein (mito-RFP) by electroporation of the mito-RFP plasmid into neurons (Fig. 1A and 1B; also see Li et al., 2014). Several improvements were made based on published designs (Cui et al., 2007; Zhang et al., 2010), including reducing the overall size of the microfluidic device and decreasing the space between the microgrooves. This improved design made it possible to assemble the microfluidic chamber onto a standard 18-mm circular coverslip and to fit the entire device in a single well of conventional 12-well cell culture dishes. Furthermore, decreasing the height of the axonal microgrooves to 5 μm allowed all mitochondria in the axonal bundles to be focused within 5 focal planes during confocal microscopy. This microfluidic chamber neuronal culture system together with a powerful confocal microscope (such as Leica SP8) allowed fast imaging with a better time resolution (at 0.3–1.5 s intervals). In addition, our microfluidic system made it possible to track most, if not all, mitochondria in the field of interest because most axonal mitochondrial (mito-RFP) signals were contained inside parallel axonal microgrooves, allowing efficient identification of mitochondria moving in anterograde or retrograde directions.

**Figure 2:**
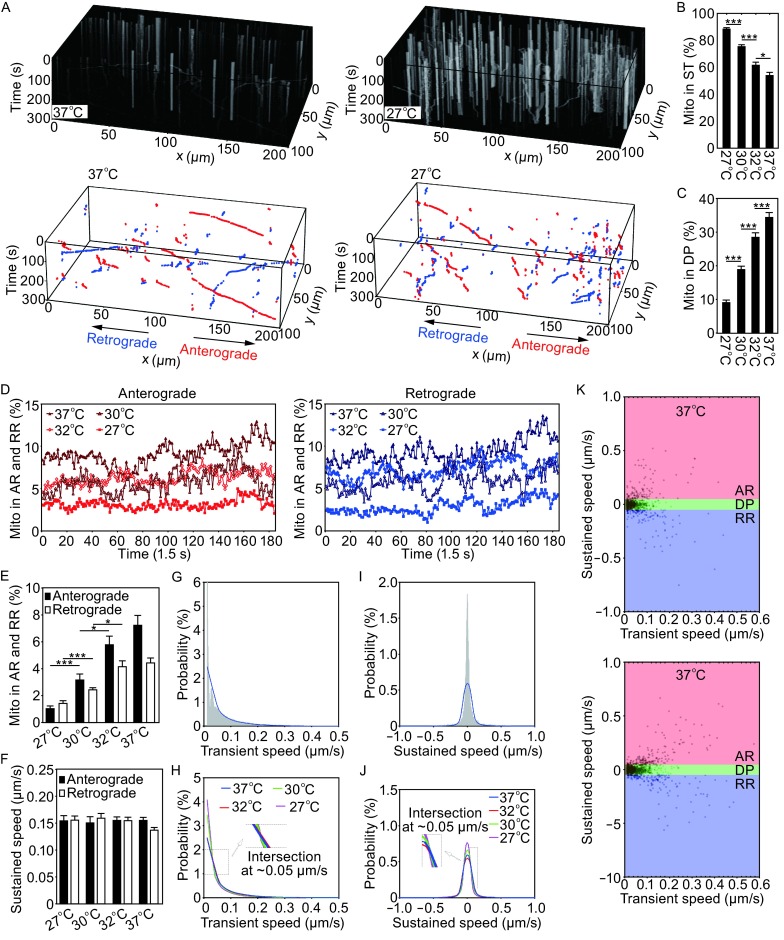
Temperature changes significantly affect axonal mitochondrial movement. (A) 3-D kymographs (top panels) and trajectories generated by MT (bottom panels). A 3-D kymograph was generated by projecting each frame [a 3-D (*xyz*) image] to a 2-D (*xy*) image using the maximum method with the *xy-t* data visualized in the 3-D kymograph. To increase the clarity of the diagram, data collected at two temperatures (37°C and 27°C) are included. In addition, only trajectories of mitochondria in AR or RR states are shown in red and blue colors respectively, without those in the DP state. See Figure S1 for complete set of data for 32°C and 30°C, which includes the DP state. (B) The percentage of mitochondria in stationary state increased as temperature went down from 37°C to 27°C. (C) The proportion of mitochondria in dynamic pause state decreased as temperature was reduced. (D) The proportion of mitochondria in different running states over time. Mitochondrial movement is illustrated by red (anterograde) and blue traces (retrograde). (E and F) Respectively, the proportion of mitochondria running in either directions (AR or RR) and the sustained speed, which can be considered equivalent to the short-term average speed. (G) Histogram of the probability distribution of transient component of speed and its corresponding regression (blue line). (H) Comparison of transient speed distribution among different temperature groups, Curves were averages of twelve image sequences. All curves intersected at ~0.05 μm/s. (I) Histogram of the probability distribution of sustained component of speed and its corresponding regression (blue line). (J) Comparison of sustained speed distribution among different temperature groups. (K) A 2-D parameter space created by calculating sustained speed and its transient speed variance. The ST state was marked in small brown color area near the origin of coordinates zero. At least 60 axonal bundles (12 image series, 5 axonal bundles per image series) from at least 4 independent microfluidic chambers were imaged for each group. At least 1000 mitochondria were identified and quantified for each group. Data represent at least 3 independent experiments [one-way ANOVA, (*P < 0.05; **P < 0.01; ***P < 0.001)]

In a typical experiment, we isolated E18 rat cortical neurons and seeded the neurons into the microfluidic chambers following electroporation with the mito-RFP plasmid to label mitochondria. The neurons were cultured for 7–9 days in the microfluidic chambers on coverslips with each chamber containing >200 axonal bundles inside the microgrooves. During real-time confocal microscopy, we set the image resolution to 512 × 1024 pixels to cover an area of 5 axonal bundles (100 μm) in width and 200 μm in length (Fig. 1B). Within this window, ~100 mitochondria were imaged and tracked simultaneously. This sample size is 5–10 times more than those in previous studies (e.g., Wang et al., 2011). Because of the small cross-section size of the microgrooves, the minimal number of *z* layers was reduced to five to cover all mitochondria within the 5 axonal bundles. In addition, our Leica SP8 confocal microscope was set at the 8 kHz fast scanning mode. These new features allowed a high scanning speed with the time for 5-layer *z*-stack imaging of multiple axonal bundles optimized to 1 s, making it possible to track moving axonal mitochondria at the time resolution of 1 Hz (2 Hz when imaging a single axonal bundle). To characterize dynamic changes of mitochondrial movement at different states (defined in Fig. 1C), we captured time-lapse 3-D images for 300 s (200 frames, 1.5 s interval, as shown in Fig. 2A). This imaging condition was experimentally optimized based on considerations of time resolution, tracking duration and quantity of mitochondrial (related to area of scanning) as well as photo-bleaching. The large quantity of mitochondria imaged simultaneously provided us with large datasets and enabled us to develop high-throughput programs to automatically track and analyze mitochondrial movement in a quantitative manner and at high efficiency.

### MitoQuant: A program package for mitochondrial movement analysis

We developed a software toolkit named “MitoQuant”, containing two image-analysis programs: the MiTracker (MT) and the motion pattern analyzer (MPA) (Fig. 1D). The mitochondrion-tracking program, MT, was designed to locate mitochondria in a 3-D image sequence and link their coordinates to 3-D trajectories (See MATERIALS AND METHODS). The MitoQuant algorithm was coded with MATLAB (R2014b). The source code and data for the method presented in this manuscript are provided in the Supplementary Information with the Software and tutorial available online at http://ese.nju.edu.cn/yogo/mq.zip. Sample data for demonstration are provided at http://ese.nju.edu.cn/yogo/sampleimage.zip.

With several parameters properly set, including average mitochondrion size, threshold for segmentation and threshold for clustering (for details see MATERIALS AND METHODS), MT was designed as a fully automated program to track mitochondria in a low SNR 3-D image sequence.

The post-processing program, MPA, was designed to extract motion pattern information from the mitochondrial trajectories that MT generated. In the MPA algorithm, we introduced a transient model for mitochondrial movement and transient velocity analysis. To improve the time resolution of the analyses, we developed a transient velocity analysis approach in MPA by computing transient component and sustained component of the velocity in short periods of time window, rather than directly calculating the average speed. The detailed description of calculation of transient velocity and sustained speed is presented in the MATERIALS AND METHODS section. In our analyses, the transient component of the speed defines the motility of mitochondria, whereas the sustained speed specifies net mitochondrial movement, equivalent to the average speed in the traditional kymographic method.

**Figure 3:**
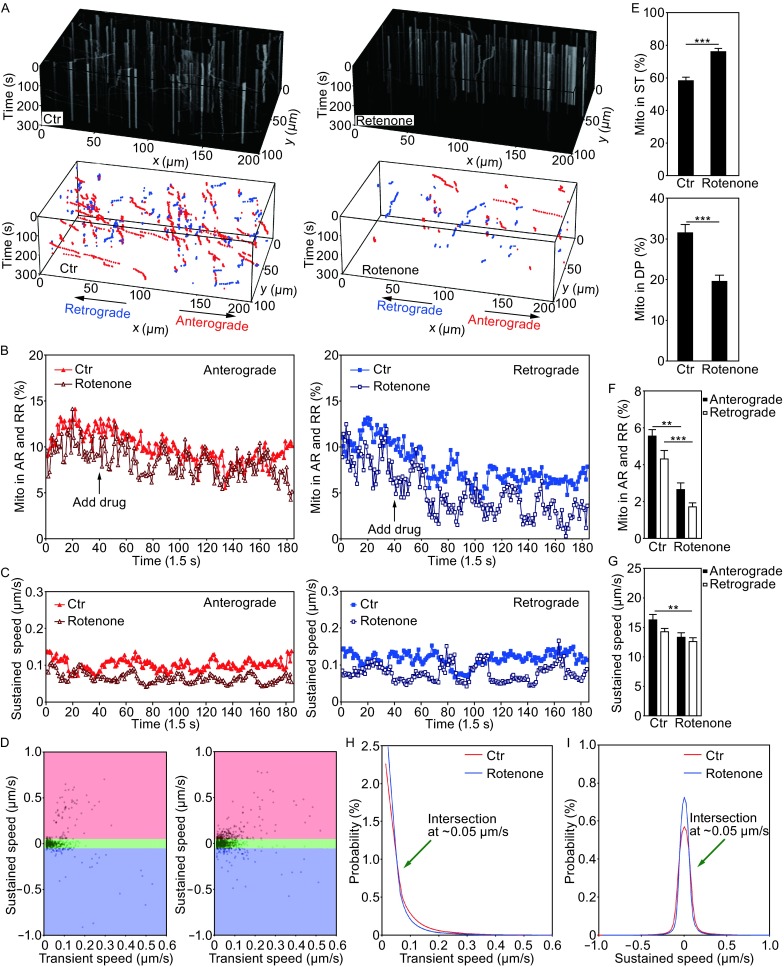
Validation of MitoQuant in quantitative analyses of axonal mitochondrial movement in rotenone treated neurons. (A) 3-D kymographs and trajectories were generated using MiTracker (MT). Neurons were treated with the control (Ctr) or Rotenone. (B) After 1 min of imaging, rotenone was added to the culture medium to the final concentration of 1 μmol/L; and real time fluorescent confocal microscopy was continued for additional 4 min. The proportion of mitochondria in running states (AR or RR) was significantly reduced following rotenone treatment. (C) Rotenone treatment did not significantly alter the sustained speed of axonal mitochondria over time. (D) A 2-D parameter space created by calculating sustained speed and corresponding transient speed. The ST state was marked in small brown color area near the origin of coordinates zero. (E) The proportion of axonal mitochondria in the ST state was significantly increased following rotenone treatment. The proportion of mitochondria in the DP state was decreased from ~28% to ~19% following rotenone treatment. (F and G) The proportion of axonal mitochondria in running states (AR or RR) and their corresponding sustained speed, respectively. The variations of sustained speed among groups were small, as compared with variations of proportional indices. (H) Distribution of transient speed of axonal mitochondria. (I) Comparison of sustained speed distribution between rotenone treated and control groups of neurons. All curves in panels (H) and (I) were average of twelve image sequences. At least 60 axonal bundles from at least 4 chambers were imaged for each group. At least 1100 mitochondria were identified and quantified for each group. Data represent at least 3 independent experiments [one-way ANOVA, (*P < 0.05; **P < 0.01; ***P < 0.001)]

Transient velocity analysis converted 3-D mitochondrial trajectories to points in a 2-D parameter space generated by two components of mitochondrial speed. In the 2-D parameter space, mitochondrial movement states were visualized and identified (see Figs. 2K and 3D), with each point in the space mapped by movement state of individual mitochondrion at one specific time point. This made it possible for MPA to perform mitochondrial motion analysis in a better time resolution than those reported by previous studies, allowing us to examine fast dynamic changes of axonal mitochondrial movement. As a result, MPA yields not only statistical indices such as the proportion of mitochondria in each movement state and the average running speed, but also indices that specify transient states of mitochondrial movement.

### A transient model for mitochondrial movement

In recent theoretical studies (Muller et al., 2008, 2010), it has been proposed that the bidirectional mitochondrial transport arises from a stochastic tug-of-war between two families of opposing motor proteins: kinesins and dyneins. The fast bidirectional movement was explained by a dynamic instability arising from the nonlinear force-dependent unbinding rate of motor protein(s), exhibiting numerous pauses and direction switches (Muller et al., 2008, 2010). This theory was supported by published experimental data (Wang et al., 2011) and by our results. Consistently, we observed that the vast majority of mitochondria travel at a constant velocity regardless of their movement directions (anterograde versus retrograde) (Fig. 1C). It should be noted that mitochondria might seem to travel at a lower velocity (curves with smaller gradient on kymographs), especially when time resolution of imaging was low. However, this was an artifact because fast stochastic switches between directions were beyond the scope of the time resolution and therefore undetectable.

We established a systematic model for axonal mitochondrial transport based on this stochastic tug-of-war principal. In this model, four movement states were defined as: stationary (ST), dynamic pause (DP), anterograde running (AR) and retrograde running (RR) (Fig. 1C). The ST was defined as the mitochondrial movement state without net linear movement or oscillatory motion. The absence of motility that mitochondria exhibited in the ST state was previously described as long-term pause (e.g., Chang et al., 2006; Kang et al., 2008). In contrast, the states of DP and both running states (AR and RR) are defined as motile or active mitochondrial states. In the DP state, mitochondria exhibit fast switching between directions of anterograde and retrograde, resulting in no net displacement but with oscillatory motion. The dynamic pause is, in general, much shorter (typically < 15 s) than the stationary state (or long-term pause) and has a greater probability to transition to running states. In both the AR and RR states, mitochondrial movement is characterized by “smooth and steady runs” at a constant velocity of approximately 1 μm/s (e.g., Ashrafi et al., 2014; Wang and Schwarz, 2009b). However, in running states, mitochondria may also exhibit fast stochastic switching between directions. In addition, the switching period could be beyond our current scope of time resolution. Therefore, the observed average velocity varied across a wide range (0.05–1.0 μm/s). It is possible that the binding ratio of two families of functionally opposite motor proteins (kinesins and dyneins) may determine the movement state of mitochondria and that the bidirectional mitochondrial movement is the result of stochastic tug-of-war of the two families of opposing motor proteins.

To analyze dynamic processes of mitochondrial movement, we introduced a transient velocity analysis method into the MPA algorithm. The transient velocity analysis was achieved by computing the sustained component and the transient component of the velocity within a short time window (see MATERIALS AND METHODS for more details). Here, “sustained speed” was considered equivalent to the short-term average speed. As a result, a 2-D parameter space, in which motion states were visualized and identified, was established by two components of velocity to define the motion states (see Figs. 2K and 3D). For instance, the state of ST was mapped to the original point of the space. On the other hand, points near the x-axis (with a low sustained speed) were defined as the DP state. This transient model of movement made it possible to quantify and analyze mitochondrial movement using systematic indices, including velocity of movement, percentage of “active” mitochondria and proportion of mitochondria in each state.

### Motion pattern analysis in the 2-D parameter space

**Figure 4:**
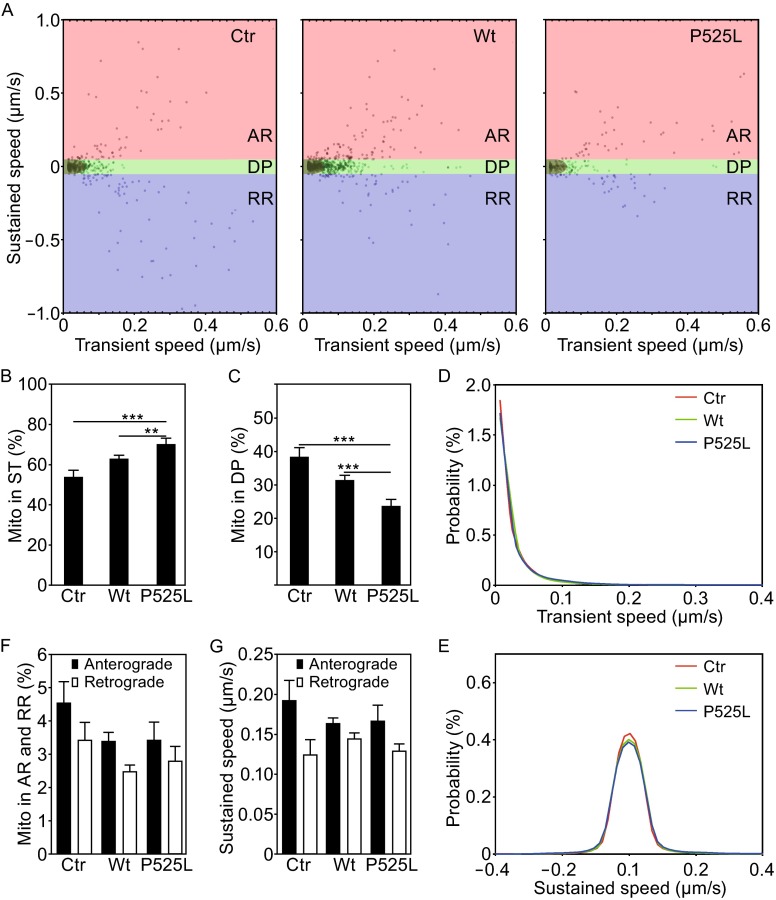
Expression of FUS protein decreases axonal mitochondrial transport in cultured mammalian neurons. (A) A 2-D parameter space was created by calculating sustained and transient component of speed of mitochondrial movement in each group of neurons expressing the vector control (Ctr), wild-type FUS (Wt) or P252L-mutant FUS (P525L) proteins. (B and C) A significant increase in the percentage of mitochondria in stationary (ST) and a significant decrease in the percentage of mitochondria in dynamic pause (DP) in axons of neurons expressing either Wt-FUS or P525L-mutant FUS as compared with the control group. (D) The regression results of alternate component of transient speed. The distribution of transient speed in different group was similar. (E) The distribution comparison of sustained speed. (F) Percentage of mitochondria in running state (AR or RR) was determined among different groups of cultured neurons. (G) Sustained speed of running mitochondria was determined in different groups of cultured neurons. At least 100 axonal bundles from at least 4 chambers were analyzed for each group. Data represent at least 3 independent experiments [one-way ANOVA, (*P < 0.05; **P < 0.01; ***P < 0.001)]

To evaluate the proposed transient velocity analysis, which was the core algorithm of MPA, we designed a series of experiments to examine axonal mitochondrial transport under different conditions, including temperature changes, drug treatment and genetic manipulation that are known to affect mitochondria (Figs. 2, 3 and 4). First, the axonal mitochondrial transport was examined at different temperatures, 37°C, 32°C, 30°C and 27°C respectively, for a period of 5 min (Fig. 2). Temperature changes alter the biophysical state of mitochondrial membranes and perturb the equilibrium among oxidative phosphorylation, electron transfer and ROS production (Borland et al., 2008). In addition, mitochondrial enzyme activities change at different temperatures. As a result, the movement patterns of mitochondria change at different temperatures. We used the mitochondrion-tracking program, MiTraker, to obtain mitochondrial trajectories from the 3-D image sequences captured by fluorescent confocal microscopy (Fig. 2A). MPA was then used for the transient velocity analysis, calculating sustained component and transient component of mitochondrial movement velocity within sliding time windows of 16 time points over 24 s (1.5 s/frame). As shown in Fig. 2K, the two components of velocity generated a 2-D parameter space in which mitochondrial movement states were intuitively visualized. Ideally, the ST mitochondrial state was identified as those points located at the origin of coordinates zero. Considering noises introduced during imaging and computing, we applied a threshold (0.05 μm/s) to both components of speed to distinguish the mitochondria at the stationary phase from those in other states. This threshold was selected because it provided the best sensitivity for our algorithm under our imaging conditions in different paradigms (See below). The same threshold was applied to the sustained speed to separate DP from both running states of AR and RR. Our data showed ~54% of mitochondria in cultured cortical neuron axons were in the ST state at 37°C. As the temperature decreased, the proportion of stationary mitochondria increased significantly (Fig. 2B, ~61% at 32°C, ~75% at 30°C and ~89% at 27°C), showing a clear reverse correlation between mitochondrial movement states and temperature changes. Our analyses indicate that >50% mitochondria are in the ST state in the neuronal axons under our culture condition and that the proportion of mitochondria in the ST state increases as temperature decreases. Consistently, the proportion of motile mitochondria including those in AR or RR running states and DP state exhibits a strong correlation with temperature changes (Fig. 2E and 2C).

To determine the threshold distinguishing the mitochondrial movement states, we fitted the histograms of sustained speed and transient speed with curves (Fig. 2G and 2I). The normalized curves described mitochondrial speed distribution (i.e. the probability of speed) at different temperatures, as shown in Fig. 2H and Fig. 2J. Interestingly, we observed that all curves intersected at ~0.05 μm/s in both sustained-speed distribution map and transient-speed distribution map (Fig. 2H and 2J). The areas (which are equivalent to proportions) under the curves in each state concurrently change in most significant manner when threshold was set to the intersection value. This provides the rationale for us to use this value (0.05 μm/s) as the threshold to define motile versus non-motile states for mitochondria, giving the algorithm the best sensitivity.

We also observed that the sustained speed distribution in different groups were very close to each other, indicating that the average mitochondrial movement speed in different groups was similar. To examine how temperature change affects the index of average speed, which was used to describe mitochondrial motion in many studies. The average speed of mitochondrial movement in each state was calculated. The results were consistent with the observation in speed distribution map that the sustained component of speed (the short-term average speed) was barely affected by temperature changes (See Fig. 2F). These data suggest that the previously defined index of the average speed had insufficient accuracy and sensitivity. Our observations also suggest that simply computing the average speed or running length, as is the case of many previous studies, may lead to a remarkable loss of algorithm sensitivity and therefore biased conclusions. As compared with those traditionally used indices, the proportion of motion states is more stable, sensitive and robust (Fig. 2B, 2D and 2F).

### Transient velocity analysis improved time resolution in mitochondrial movement analysis

The transient velocity analysis made it possible for us to use MPA to analyze mitochondrial movement at a better time resolution, because the analysis was performed at each sampling point with a sliding window, instead of using one entire time period as in previous studies. In the experiment with temperature changes, the dynamics of mitochondrial movement exhibited temporal stability. Indices such as the proportion of mitochondria in different states and the sustained speed of AR and RR remained stable over time (Fig. 2F).

Next, we used a rotenone-treatment paradigm to evaluate the capability of MPA in fast dynamic analyses (Fig. 3). Rotenone is a high-affinity non-competitive inhibitor of mitochondrial ATP synthase complex I, affecting mitochondrial ATP production (Fukami, Yamamoto and Casida Fukami et al. 1967; for recent reviews see Sanders and Greenamyre, 2013). Rotenone has been widely used as mitochondrial inhibitor by reducing membrane potential and inducing mitochondrial damage. Several different mechanisms may contribute to the reduced axonal mitochondrial transport following rotenone treatment (Greenamyre et al., 2003; Radad et al., 2006). In addition to binding to complex I, rotenone also binds to microtubules and disrupts axonal transport (Srivastava and Panda, 2007). As a result, rotenone treatment significant reduces mitochondrial motility.

We captured the entire image sequences of axonal mitochondria for a period of 5 min in both the control- and rotenone-treated neurons (Fig. 3A and 3B). At the time point 40 (corresponding to 60 s, Fig. 3B) after the start point of imaging, rotenone was applied to axonal chambers in neuronal cultures, and the imaging was continued to time point 200 (corresponding to 300 s). Figure 3B demonstrates the index changes over time. MT and MPA were used to analyze data and calculate statistical indices (Fig. 3B–G). Our data show that rotenone treatment significantly increased the percentage of mitochondria in ST state, but reduced the proportion of mitochondria in either DP or running states (AR or RR; Fig. 3B, 3E and 3F). The average speed of either AR or RR was also reduced by rotenone treatment (Fig. 3C, 3D and 3G). However, change in the average speed index was small, in comparison with that of proportion indices, suggesting that the index of average speed had lower algorithm sensitivity. The normalized curves for mitochondrial speed distribution (i.e. the probability of speed) of the control and rotenone treated groups are shown in Fig. 3H and 3I. Consistently, the curves intersected at ~0.05 μm/s in both sustained-speed distribution map and transient-speed distribution map (Fig. 3H and 3I). Our data indicate this transient motion analysis efficiently captured the dynamic process of mitochondrial movement and showed sufficient time resolution to monitor dynamic mitochondrial movement in live axons.

### MitoQuant analyses reveal defects in axonal mitochondrial transport in neurons expressing FUS proteins *in vitro* and *in vivo*

Defective mitochondrial biogenesis and transport have been associated with a range of neurodegenerative diseases (see Mattson et al., 2008; Wallace 2013; Devine et al., 2015; Tourtellotte, 2015). Our recent work has shown that fused in sarcoma/translocated in liposarcoma (FUS/TLS or FUS), a nuclear RNA binding protein, interacts with and targets mitochondria (Deng et al., 2015). This prompted us to carefully examine effects of wild-type and ALS-mutant FUS on axonal mitochondrial transport.

We expressed FUS in cultured mouse cortical neurons by co-transfecting Mito-Red with either a control GFP vector or a plasmid expressing GFP-tagged FUS protein and then cultured transfected neurons in the microfluidic chambers for 9 days. The cultured neurons expressing Mito-Red together with Wt- or an ALS-mutant P525L-FUS were then used for time-lapse fluorescent confocal microscopy. At the imaging stage, twelve 3-D image sequences were captured for each group: the control, Wt-FUS or P525L-mutant FUS. The total sampling number for each group (number of mitochondria) was larger than 1000. MitoQuant was then used for high throughput analyses of axonal mitochondrial transport in neurons expressing the vector control, Wt-FUS or P525L-FUS (Fig. 4A–G). A 2-D parameter space was created for the corresponding group by calculating the transient and sustained components of mitochondrial movement speed (Fig. 4A). Analyses by MPA indicate a significant increase in the proportion of mitochondria in the ST state and a significant decrease in the proportion of mitochondria in the DP state in neurons expressing either Wt- or P525L-mutant FUS as compared with the control group (Fig. 4B and 4C). The distribution of transient speed or sustained speed of mitochondrial movement in neurons expressing either Wt- or P525L-mutant FUS was similar to that in the control group (Fig. 4D and 4E). The percentage of mitochondria in either anterograde or retrograde running states (AR or RR) was moderately decreased in FUS-expressing neurons (Fig. 4F), whereas changes in the sustained speed of running mitochondria were not significant (Fig. 4G). These data have revealed previously unknown activity of FUS in affecting axonal mitochondrial transport.

**Figure 5:**
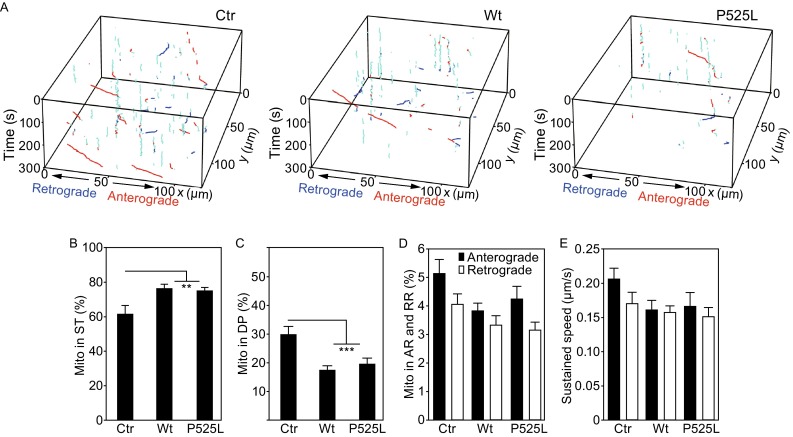
Expression of FUS protein significantly affects axonal mitochondrial transport in motor neurons of transgenic flies expressing FUS. (A and B) A significant increase in the proportion of mitochondria in stationary (ST) state and a significant decrease in the percentage of mitochondria in dynamic pause (DP) state in neurons expressing either Wt- or P525L-mutant FUS, as compared with neurons in the control group. (C) The percentage of axonal mitochondria in running states (AR or RR) among different groups. (D) The sustained speed of running mitochondria was determined in different groups of flies. At least 20 axonal bundles (containing 100–200 axons) were analyzed for each group. Data represent at least 3 independent experiments [one-way ANOVA, (*P < 0.05; **P < 0.01; ***P < 0.001)]

To further examine FUS activity *in vivo*, we used our previously established fly model for FUS proteinopathy (Chen et al., 2011; Deng et al., 2015). We crossed mitoGFP into the transgenic flies expressing human Wt- or P525L-mutant FUS to track mitochondrial movement in motor neuron axons of these transgenic flies. We then used MitoQuant to perform an automated analysis of axonal mitochondrial movement in the live fly motor neurons. Our data show that the proportion of moving mitochondria was significantly reduced in flies expressing either Wt- or P525L mutant- FUS as compared with the control flies (Fig. 5A–C). Both the proportion of mitochondria in either anterograde or retrograde running states and their corresponding sustained speed of movement were also moderately reduced (Fig. 5D and 5E). These data are consistent with that obtained from cultured mammalian neurons, both supporting that increased FUS expression reduces axonal mitochondrial transport. Previous studies have shown increased FUS expression in patients affected by frontotemporal lobar degeneration associated with FUS (FTLD-FUS) (Mackenzie et al., 2011; Sabatelli et al., 2013; Deng et al., 2015). Consistently, transgenic animals overexpressing the human FUS protein recapitulate critical pathological and clinical features of FUS proteinopathy (e.g, Huang et al., 2011; Chen et al., 2011; Lanson et al., 2011). Together, our results suggest that reduced axonal mitochondrial transport may contribute to pathogenesis of FUS proteinopathy.

## DISCUSSION

### An efficient imaging method with high throughput analysis software

In this study, we developed a microfluidic chamber culture method together with analyses software for efficiently imaging and quantitatively analyzing axonal mitochondrial transport. It should be noted that in many published studies, axonal mitochondrial transport had been examined in individual isolated axons of monolayer-cultured neurons. This may not best reflect physiological conditions in which the majority of axons exist as axonal bundles rather than isolated individual axons. To image axonal mitochondrial transport in axonal bundles, we used a micro-fluidic chamber system for culturing neurons that we previously established (Li et al., 2014) and optimized real-time fluorescent confocal microscopy on a Leica SP8 system. Our newly established method made it possible to image a large number of mitochondria simultaneously at a time resolution up to 1 Hz. In our experiments, a single 5-min 3-D (*z*-stack) image procedure allowed detection of ~100 mitochondria for quantitative analyses. It was a significant improvement as compared with the published methods. The traditional imaging procedure for the dynamic mitochondrial transport was extremely time-consuming. Sample size was therefore limited, making the conclusion less objective. For instance, in our validation experiment, at least 60 axonal bundles (each containing approximately 5~10 axons) were imaged for each group. At least 1000 mitochondria were identified and quantified. More importantly, higher time resolution allowed fast dynamics of mitochondrial movement to be captured and analyzed.

Our MitoQuant toolkit was designed to contain two image-analysis programs (MT and MPA) to automatically process and analyze a large amount of imaging data. This software can handle not only neurons cultured in the microfluidic chambers in a high throughput manner, but also neurons *in vivo* that are imaged in a conventional way. We validated our algorithm in live drosophila motor neurons. The results were consistent with that obtained from the cultured neurons.

Overall, our method described in this study has several unique aspects, making it more powerful and efficient than the previously published ones. First, in comparison to the manual or semi-automatic recognition of mitochondria in the traditional methods, our MiTracker program offers increased efficiency and accuracy. Second, a 3-D trajectory map is generated by MiTracker, instead of 2-D kymographic data output, making the analyses more objective, less biased and with increased accuracy as well as sensitivity. Third, the transient and sustained components of movement velocity are both utilized to characterize mitochondria in movement, offering a more stable, sensitive and robust approach to analyzing moving mitochondria than previously published methods.

Because of the critical role of mitochondrial transport in neuronal function and extensive involvement of mitochondrial transport in a large number of human disorders, including neurodegenerative diseases, our newly developed method will be highly useful for studying human diseases associated with mitochondrial transport, in particular, in cellular and animal models for these diseases.

### The mitochondrial movement patterns

To characterize mitochondrial movement patterns, we established a systematic motion model to define mitochondrial movement states. With the increased time resolution for imaging and improved algorithms, we identified a movement state for axonal mitochondria that had not been described previously. We name this movement state as “dynamic pause” and use it to describe mitochondria with a low sustained speed but a high transient component of velocity. In the previously published work, these mitochondria in DP state were regarded as stationary. Such a mis-assignment may introduce increased bias to mitochondrial analyses. Indices such as moving speed, running length, and pausing frequency that were used to characterize mitochondria movement in the published studies, may therefore suffer from losses of sensitivity and objectiveness. Our analyses suggest that mitochondria in the DP state may have higher probability to transition into running states than those in true stationary state. Our data indicate that the proportion of mitochondria in the DP state is more sensitive to factors that affect mitochondrial function, including temperature change, rotenone treatment and expression of mitochondrion-modulatory gene(s). Therefore, in our motion model, the “active” states of mitochondria include both running state and DP state. This definition of active mitochondrial movement states may provide additional robustness in analyzing highly complex and heterogeneous populations of mitochondria under different developmental or disease conditions.

### Transient velocity analysis for studying dynamics of mitochondrial movement

In developing the core algorithm of MitoQuant, we adopted the stochastic tug-of-war explanation of mitochondrial bidirectional motion. A number of theoretical and experimental studies support this model for mitochondrial movement. Our data indicate mitochondria travel at a constant velocity regardless of their direction of movement (anterograde or retrograde). It was confusing that sometimes mitochondria may exhibit a lower apparent movement velocity (e.g. Fig. 1C). Our high time-resolution analyses indicate that such a “slowdown” in mitochondrial movement was an artifact from analyses of incomplete data. The fast stochastic switching in mitochondrial movement may exceed the temporal resolution of the imaging and analyses. Furthermore, our data suggest that simply calculating the frequency of switching movement directions may result in increased biases, because the motile mitochondria in live cells may switch their movement directions stochastically and at a high frequency beyond the scope of time resolution. In addition, at a low temporal resolution, boundaries of mitochondrial movement states were often obscure, leading to incorrect classification of mitochondrial movement states. For example, motile mitochondria in the dynamic pause state might be incorrectly identified as stationary, resulting in possibly biased conclusions.

To objectively quantify mitochondrial movement, we introduced transient velocity analysis into our MPA algorithm. This transient model made it possible to characterize the fast dynamics of mitochondrial movement with systematic indices. Our newly developed method for mitochondrial imaging and analyses method will be highly useful for studying human diseases involving mitochondrial damage.

It should be noted that our newly developed program can be applied to not only studying mitochondrial movement in cultured neurons but also for analyzing movement or transport of other types of particles including neuronal vesicles, intracellular organelles such as peroxisomes and other macromolecular machineries (such as RNA-protein aggregates). Therefore, this program will be highly useful and versatile for investigating dynamic movement of biological complexes and for understanding molecular defects underlying a wide range of human diseases involving mitochondrial trafficking or formation of aberrant aggregates containing either proteins or nucleic acids.

Considering the genetic heterogeneity and complexity in regulation of mitochondrial biogenesis and movement, future studies will be necessary to include factors such as mitochondrial fission and fusion when developing more robust and sensitive programs for mitochondrial movement. In addition, further work is needed to resolve issues associated with phototoxicity and photo-bleaching. This will be accomplished when more photostable fluorophores are used and even higher time resolution becomes achievable.

## MATERIALS AND METHODS

### Microfluidic chamber fabrication

We followed established protocols to fabricate microfluidic chambers (Cui et al., 2007; Li et al., 2014). Briefly, masks were designed with AutoCAD (Autodesk). Masters consisting of 2 layers of photosensitive epoxy SU-8 (SU-8 2005 and SU-8 2010, respectively, from MicroChem) patterns were prepared by standard photolithography with a mask aligner (MJB4; Süss MicroTec) in a clean facility. The first layer of SU-8 (5 μm in depth) contained the microgrooves (5 μm in height; 5 μm in width), fabricated with a high-resolution chromium mask, whereas the second layer of SU-8 (100 μm in depth) contained the compartments and central flow channels, fabricated with a printed transparency mask. Replica molding of polydimethylsiloxane (PDMS; Dow Corning) was performed to obtain the elastic microfluidic chambers. The cell body and growth cone compartments were made using punchers (Harris Uni-Core; Ted Pella, Inc.) of 5 mm in diameter, and the inlet and outlet wells of the central flow channels were made using punchers of 3 mm in diameter.

### Primary neuronal culture and electroporation

Cortices from E18 rats (Sprague-Dawley) were dissected and dissociated as previously described (Guo et al., 2011; Deng et al., 2015). Briefly, animals were euthanized, and embryos were removed from the abdomen. Cortices were dissected, placed in ice-cold Hanks’ balanced salt solution medium (Invitrogen) and dissociated with papain (Sigma) for 15 min at 37°C. Neurons were resuspended in Neurobasal medium supplemented with 2% B27 (Gibco/Life Technologies), 0.5 mmol/L L-glutamine, and penicillin/streptomycin.

For DNA plasmid transfection, dissociated cortical neurons were electroporated with DNA plasmids using the Amaxa Nucleofector apparatus. Briefly, 5 million cells were resuspended in Nucleofector solution containing 3 μg of plasmid DNA, then immediately zapped in the Nucleofector using program O-03.

After electroporation, the cells were resuspended in neuronal culture medium and plated into microfluidic chambers attached to coverslips or glass bottom culture dishes that were coated with 200 μg/mL poly-D-lysine. The cells were cultured at 37°C and 5% CO_2_, and half of the medium was changed every 72 h.

### Live cell confocal microscopy

We used an inverted laser-scanning confocal microscope adapted for live cell imaging (Leica SP8). Mitochondria were visualized through a 40× oil immersion objective. The microscope incubator was set at 37°C and 5% CO_2_. Images were collected at the resolution of 512 × 1024 pixels to cover 5 axonal microgrooves, and images were captured every 1.5 s over a total imaging time of 5 min. The serial *z*-stacks were acquired every 0.5–1 μm, which was sufficient to cover all mitochondria inside axon bundles within 5 axonal microgrooves. Precaution should be taken to avoid overexposure; and the same imaging conditions should be used for different groups of samples.

### MitoQuant program package and its parameter setting

The MitoQuant program package contains two components, MiTracker and MPA (Motion Pattern Analyzer), along with other functions such as image importing and results exporting. The MiTracker locates mitochondria in a 4-D image sequence (*xyz-t*) and links their coordinates to 3-D trajectories. A 3-D kymograph was generated by projecting each frame [a 3-D (*xyz*) image] to a 2-D (*xy*) image using a maximum method. The maximum method, which determines the output value by finding the maximum along z axis, is commonly used to convert a z-stack image into a 2-D (*xy*) image. A 3-D kymograph was then created by converting the time axis (t) to the third dimension with the xy-t data visualized.

MPA is designed to extract motion pattern information from the mitochondrial trajectories exported by MiTracker and to generate the statistical results based on the transient model of mitochondrial movement. For both the MiTracker and the MPA, users are required to set only a few parameters, including mitochondrial size range, threshold for segmentation, threshold for clustering and window size for transient analysis. Algorithm details are described in next subsection.

It is necessary to set the range of and average diameter of mitochondria so that a particle enhancement filter can be properly set. Users may measure this diameter in *z*-stack image by using ImageJ and input the value in pixels. The value may be adjusted depending on neuronal types and culture conditions, between 5 pixels and 13s pixel. In a typical experiment in our study, the average diameter was set at 7 pixels. The MiTracker will automatically generate several different scales of Haar windows to cover possible dimension range of mitochondrial particles (Yang et al., 2010; Yang et al., 2012). Another important parameter for MiTracker is the segmentation threshold. Our program provides an interactive interface, allowing users to adjust the threshold visually to obtain the best segmentation. Moreover, users are allowed to set independent thresholds for frame at different time points, because the threshold may vary due to significantly changing signal-to-noise ratios of the image sequences over time because of the imaging conditions.

In the MPA program, a threshold also should be set for clustering, to distinguish the mitochondria at the stationary phase from those in other states. We set the threshold as 0.05 μm/s by the observation that all curves intersected at ~0.05 μm/s in both sustained-speed distribution map and transient-speed distribution map. In our experiment, this value gives the algorithm the best sensitivity. For instance, as shown as Fig. 3I, the areas (equivalent to the proportion of mitochondria in each state) under the curves in the DP or AR state concurrently changed in a most significant manner only when threshold was set to 0.05 μm/s.

The transient analysis window size has to be $2^{k} , k \in R$. The typical value may vary between 8 and 32. In our experiment, the windows size was set to 16 points, which was a compromise between temporal resolution and speed resolution under our imaging conditions.

### Tracking mitochondria in 3-D image sequence by MiTracker

**Figure 6:**
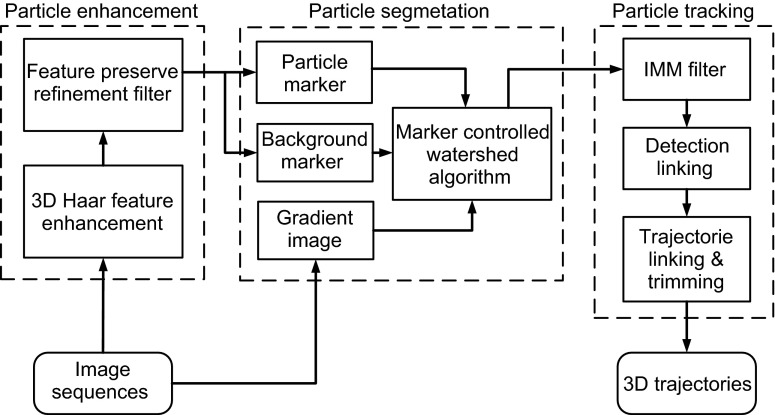
A flow chart to illustrate detailed processes of the MiTracker algorithm. There are three major components: the particle enhancement, the particle segmentation and the particle tracking. The algorithm details are described in the MATERIALS AND METHODS section about tracking mitochondria in 3-D image sequence

The algorithm of MiTracker consists of three major steps: “Particle Enhancement”, “Particle Segmentation” and “Particle Tracking” (See the flow chart illustrated in Fig. 6). To increase signal-to-noise ratio (SNR) of the image sequences, a series of pre-processing protocols were performed in the “Particle Enhancement” step. First, we mapped the original gray scale image to a feature space called a “particle probability map”, using a particle filter with Haar-like features (Yang et al., 2010; Yang et al., 2012). A non-linear filter called “Feature Preserved Non-Local Mean (FP-NLM) filter” (Feature Preserve Refinement Filter) (Yang et al., 2010; Yang et al., 2012) was then used to enhance SNR of the images. The refinement filter was used to establish a non-local statistic method capable of enhancing particle-like (mitochondria) features in images, which suffered from severe noise contamination. We then extended the filter to a 3-D version and create an algorithm of FP-NLM with 3-D Haar-like features (Yang et al., 2010; Yang et al., 2012), taking into consideration the morphological parameters of mitochondria. In the “Particle Segmentation” step, we developed a coarse-to-fine approach to identify mitochondria from the confocal image based on Particle Probability (PP) map (Buades et al., 2005). The PP maps were used to coarsely estimate particle regions. Then, a marker-controlled watershed algorithm was used to segment the mitochondria from the background, requiring one simple decision threshold to be set properly. It should be noted that the marker-controlled watershed algorithm, rather than the original watershed algorithm, was used and that the particle enhancement filter was used to generate foreground marker and background. Thus, our algorithm will not introduce an artificial increase or decrease in segmented mitochondria. The MiTracker allows users to fine tune the threshold by offering a graphic interface. The foreground (particle) and background markers were determined by the coarse estimation step. In the “Particle Tracking” step, an interacting multiple mode (IMM) filter (Yang et al., 2012) was used to make prediction of particle state, taking into consideration of position and intensity of the particles. The IMM filter, a significant improvement of the Kalman filter (Kalman, 1960), was used to differentiate multiple motion patterns. IMM is capable of selecting and switching to the best motion model by evaluating the posterior probabilities of the different motion models. In this study, the particle (mitochondrion) movement consisted of three models: random motion, the first-order linear motion and the second-order linear motion. By assigning the mitochondrial detection events in each frame to existing trajectories, the detection events were linked into trajectories frame by frame. The assignment progress was achieved by matching the measurements (the mitochondrial position and morphology in current frame) with their prediction made by the IMM filter. To improve the integrity of trajectories, a trajectories linking and trimming process was designed to remove the fragmental tracks and link short tracks into longer ones. 3-D trajectories were exported following detection linking and trajectory linking/trimming. More details about the MiTracker are described in the Supplementary program package and its Tutorial.

### The transient velocity analysis

The 3-D trajectories (Fig. 2A) can be described as serial of positions ${\text{p}}_{i}$. Thus, the real time velocity of mitochondria is defined as,

$$v_{i} = ({\text{p}}_{i-1}-{\text{p}}_{i} )/\varDelta t$$

Here $\varDelta t$ is temporal interval of 3-D images. Instead of calculating the average speed directly, we introduced transient velocity analysis to MPA by computing sustained component and transient component of the velocity in a short period of time window. This significantly improved time resolution of our analysis. To calculate the sustained speed, we first fitted positions ${\text{p}}_{i}$ in a short-term temporal window with a straight line in such a way making the sum of squared residuals of the model as small as possible. Then, let the sustained speed $v_{i}^{sustain}$ equal to the slope of the fitting line. The transient speed is determined by

$$v_{i}^{transient} = \frac{1}{N}\mathop \sum \nolimits v_{i}^{sustain} - v_{i}$$

Here *N* is the windows size, which is optimized for our setting to 16 in this study (See parameter setting describe before). The sustained speed is considered to be equivalent to the average speed, which specifies net mitochondrial movement. However, the transient component of the speed defines the motility of mitochondria. As a result, a 2-D parameter space, in which motion states were visualized and identified, was established by the two components of velocity to identify motion states (Figs. 2K and 3D). This made it possible for us to use MPA to analyze dynamic progress of mitochondrial movement.

## Electronic supplementary material

Below is the link to the electronic supplementary material.

Supplementary material 1 (PDF 3430 kb)
