# Supplementary material for: A new method for quantifying mitochondrial axonal transport
Source: Protein Cell. 2016 May 25;7(11):804–19. doi: 10.1007/s13238-016-0268-3 (PMC5084152; doi:10.1007/s13238-016-0268-3)

A new method for quantifying mitochondrial axonal transport

Mengmeng Chen[#,1a], 2, Yang Li[#,1b], 2, Mengxue Yang [1a], 2, Xiaoping Chen[2], Yemeng Chen[1b], Fan Yang[1b], Sheng Lu[1b], Shengyu Yao[2], Timothy Zhou[2], Jianghong Liu[1c], Li Zhu[1c], Sidan Du[*,1b] and Jane Y. Wu[*,2]

[1a] University of Chinese Academy of Sciences, Beijing, China; [1b]School of Electronic Science & Engineering, Nanjing University, Nanjing, China; [1c] State Key Laboratory for Brain & Cognitive Science, Institute of Biophysics, Chinese Academy of Sciences; [2] Department of Neurology, Center for Genetic Medicine, Lurie Cancer Center, Northwestern University Feinberg School of Medicine, Chicago, USA.

Supplementary Information

Figure S1. 3-D kymographs and trajectories generated by MiTracker (MT) for quantitative analyses of mitochondrial movement of neurons cultured at different temperatures from 37°C, 32°C, 30°, and 27°C. This figure corresponds to Figure 2A in the text, and is labeled in a similar manner, with more detail included here to show that a significant percentage of mitochondria were in the dynamic pause (DP) state, indicated in cyan.

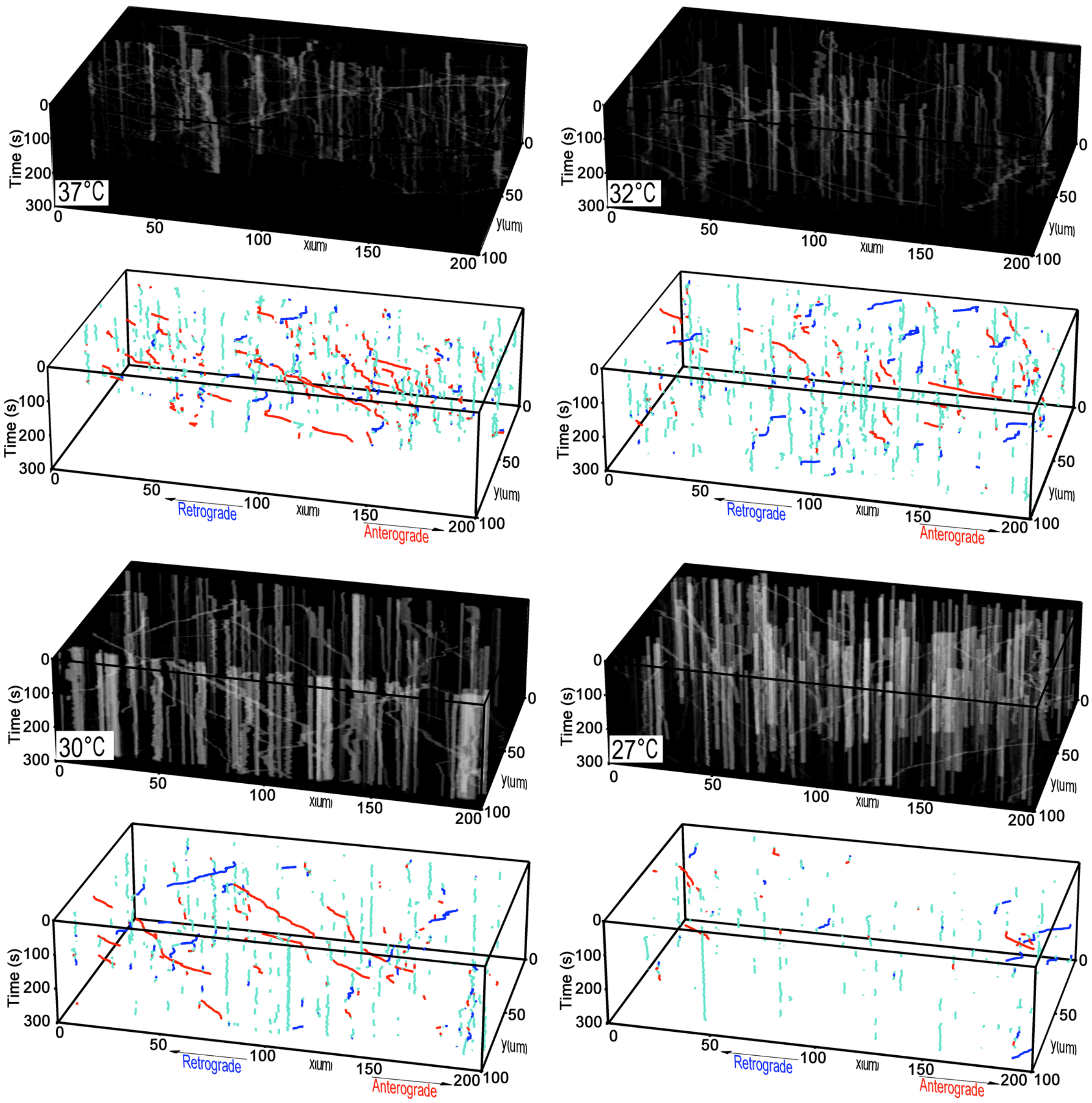

Figure S2. A) 2-D parameter space was created by calculating the sustained speed and the transient speed of mitochondrial movement in neurons cultured at different temperatures (27°C to 37°C). This includes that data shown in Figure 2K. Datasets at all four temperatures are shown to have a clearer comparison. The state of AR, RR and DP are marked in pink, purple and light green respectively. Please note the small area of brown color near the origin of the coordinate zero is the ST state.

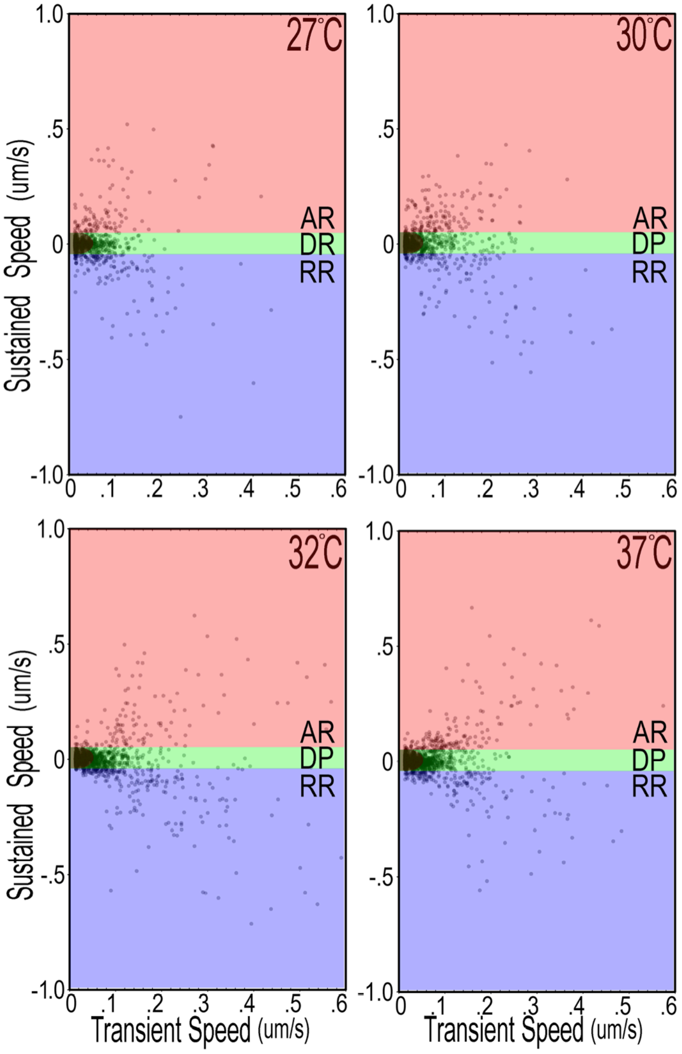

Figure S3. Comparison of distribution of transient speed and sustained speed of mitochondrial movement in cortical neurons cultured at different temperatures (27°C to 37°C). To have a clearer comparison, Datasets at all four temperatures are shown, including that in Figure 2G-2J. At least 60 axonal bundles were imaged for each group. More than 1100 mitochondria were identified and quantified for each group. Every curve represents the average of twelve image sequences. Data represent 4 independent experiments.

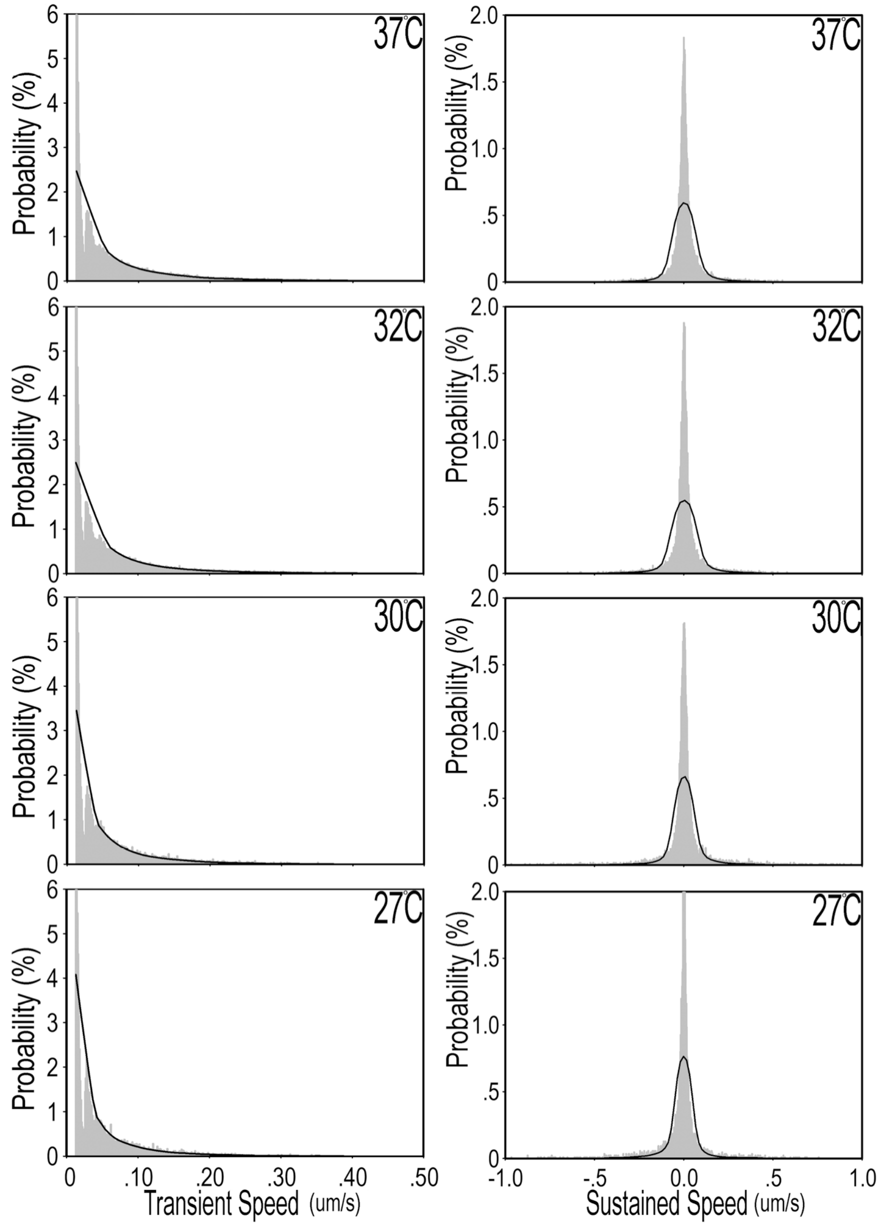

Figure S4. Distribution analyses of transient speed and sustained speed in axonal mitochondria in the presence of the mitochondrial inhibitor rotenone. (A) Comparison of transient speed distribution of axonal mitochondria between the control and rotenone treated neurons. (B) Comparison of sustained speed distribution between the control and rotenone treated groups. At least 60 axonal bundles were imaged for each group. At least 1100 mitochondria were identified and quantified for each group. Every curve represents the average of twelve image sequences. Data represent 4 independent experiments.

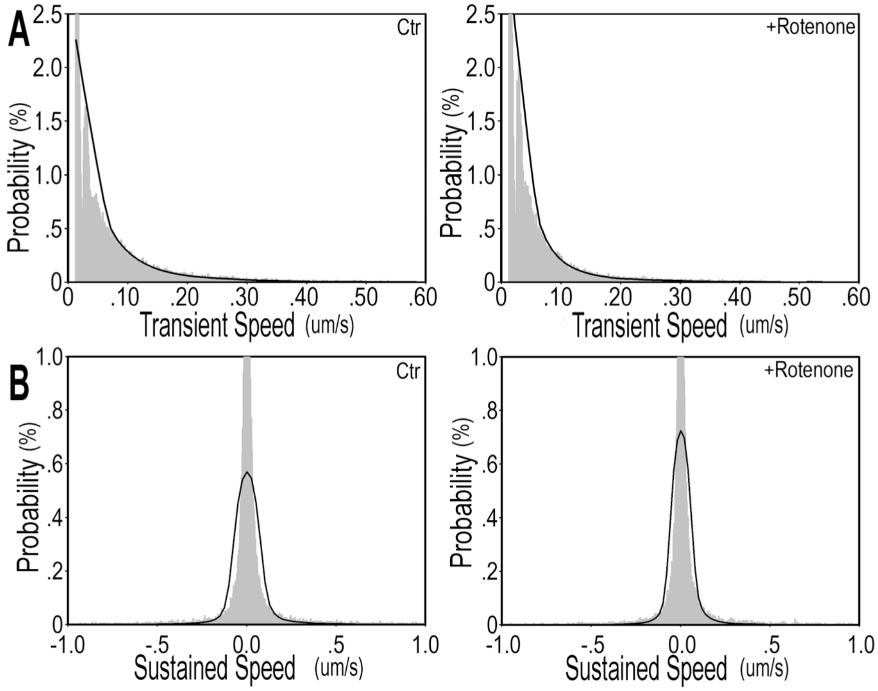

Figure S5. (A) Comparison of distribution of transient speed of mitochondrial movement among different groups cortical neurons expressing the vector control (Ctr), or Wt-FUS or P525L-mutant FUS. The curves represent regression results of the transient speed histograms. The distribution of transient speed was similar in Wt-FUS group or P525L-mutant FUS group to that in the control group. (B) Comparison of distribution of the sustained speed of mitochondrial movement among different groups. At least 60 axonal bundles were imaged for each group. At least 1100 mitochondria were identified and quantified for each group. Every curve represents the average of twelve image sequences. Data represent 4 independent experiments.

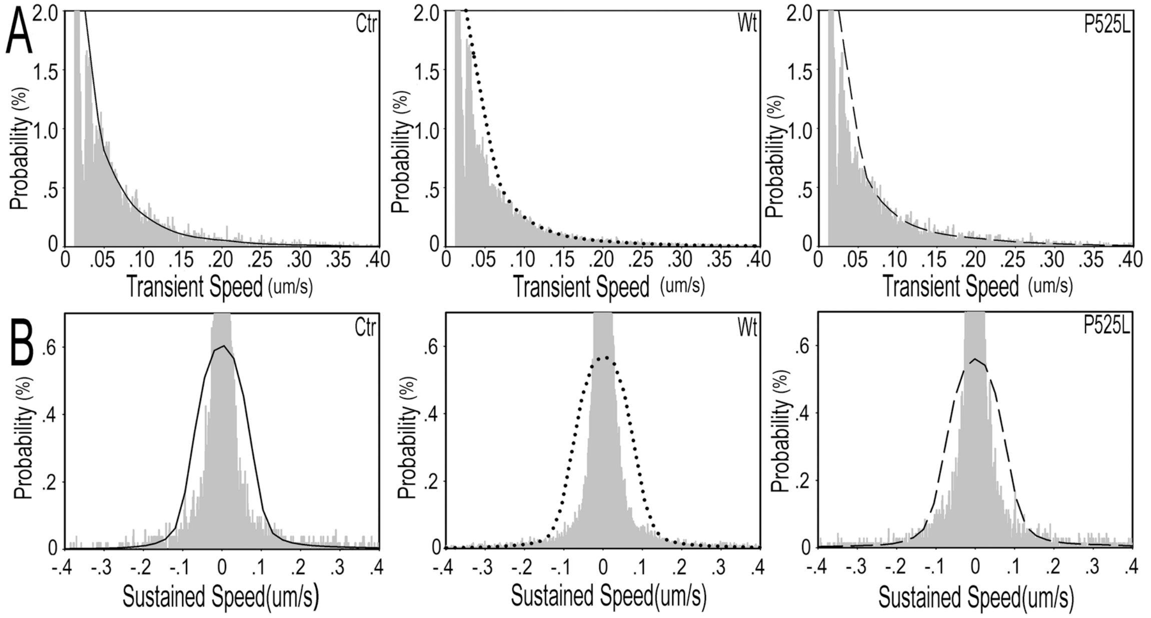

Figure S6. Confocal microscopic images of axonal mitochondria. Cortical neurons were co-transfected with plasmids expressing Mito-Red together with the GFP control (Ctr), Wt-FUS-GFP (Wt) or P525L-mutant FUS-GFP (P525L) plasmids before being seeded into the microfluidic chambers shown in Figure 1A. Fluorescent confocal microscopy was used to image mitochondrial movement inside axonal bundles as described above. Representative images are shown for the corresponding groups of neurons.

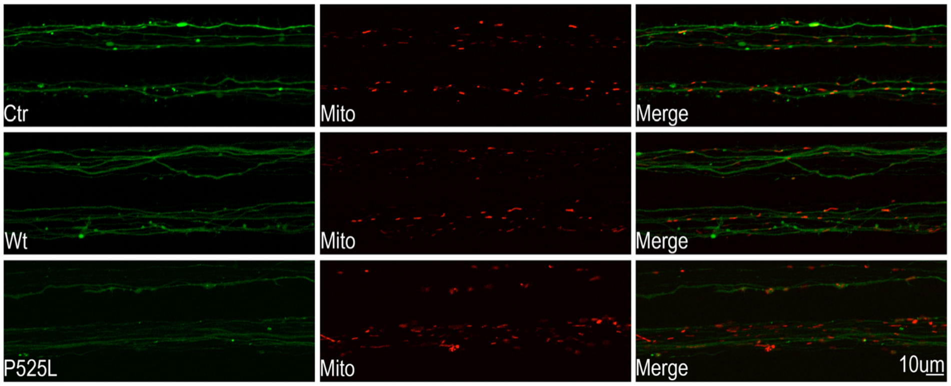

Figure S7. An example of fluorescent and bright field images of axonal bundles in the microfluidic culture. Because the PDMS device was attached to the coverslip, the images were not as sharp.

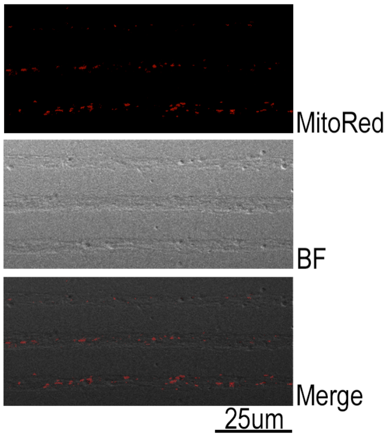

Supplement: Supplementary file 1 — Supplementary material 1 (PDF 3430 kb) [file 13238_2016_268_MOESM1_ESM.pdf]
